# Combination of Fish Oil and Selenium Enhances Anticancer Efficacy and Targets Multiple Signaling Pathways in Anti-VEGF Agent Treated-TNBC Tumor-Bearing Mice

**DOI:** 10.3390/md19040193

**Published:** 2021-03-29

**Authors:** Chih-Hung Guo, Simon Hsia, Chieh-Han Chung, Yi-Chun Lin, Min-Yi Shih, Pei-Chung Chen, Guoo-Shyng W. Hsu, Ciou-Ting Fan, Chia-Lin Peng

**Affiliations:** 1 Micronutrition and Biomedical Nutrition Laboratories, Institute of Biomedical Nutrition, Hung-Kuang University, Taichung 433, Taiwan; htjh20216@hk.edu.tw (C.-H.C.); yichunlin1021@hk.edu.tw (Y.-C.L.); smyyoga@hk.edu.tw (M.-Y.S.); 2 Taiwan Nutraceutical Association, Taipei 105, Taiwan; dr.hsia@nutraceutical.org.tw (S.H.); chenpc@sunrise.hk.edu.tw (P.-C.C.); olivia@nutraceutical.org.tw (C.-T.F.); jacquelin_peng@nutraceutical.org.tw (C.-L.P.); 3 Human Ecology College, Fu Jen Catholic University, New Taipei City 242, Taiwan; 002613@mail.fju.edu.tw

**Keywords:** antitumor mechanisms, Avastin, bevacizumab, fish oil, mice, selenium, TNBC

## Abstract

Fish oil (FO) and selenium (Se) possess antiangiogenic potential in malignant tumors. This study aimed to determine whether combination of FO and Se enhanced treatment efficacy of low-dose antiangiogenic agent Avastin (bevacizumab) in a dose-dependent manner and targeted multiple signaling pathways in triple-negative breast cancer (TNBC)-bearing mice. Randomized into five groups, mice received treatment with either physiological saline (control), Avastin alone, or Avastin in combination with low, medium, and high doses of FO/Se. The target signaling molecules for anticancer were determined either by measuring protein or mRNA expression. Avastin-treated mice receiving FO/Se showed lower tumor growth and metastasis than did mice treated with Avastin alone. Combination-treated mice exhibited lower expressions in multiple proangiogenic (growth) factors and their membrane receptors, and altered cytoplasmic signaling molecules (PI3K-PTEN-AKT-TSC-mTOR-p70S6K-4EBP1, Ras-Raf-MEK-ERK, c-Src-JAK2-STAT3-TMEPAI-Smad, LKB1-AMPK, and GSK3β/β-catenin). Dose-dependent inhibition of down-stream targets including epithelial-to-mesenchymal transition transcription factors, nuclear cyclin and cyclin-dependent kinases, cancer stem cell markers, heat shock protein (HSP-90), hypoxia-inducible factors (HIF-1α/-2α), matrix metalloprotease (MMP-9), and increased apoptosis were observed. These results suggest that combination treatment with FO and Se increases the therapeutic efficacy of Avastin against TNBC in a dose-dependent manner through multiple signaling pathways in membrane, cytoplasmic, and nucleic targets.

## 1. Introduction

Triple-negative breast cancer (TNBC) is an aggressive, invasive, and metastatic subtype of breast cancer [1]. Compared with non-TNBC patients, TNBC patients have a poorer prognosis, higher resistance to anticancer agents, and higher rates of early cancer recurrence that contribute to rapid growth of distant metastasis and shorter overall survival [2]. The treatment options for TNBC are still limited by a lack of specific targets and resistance to conventional chemotherapy [1].

TNBC patients have significantly higher levels of vascularization and vascular endothelial growth factor (VEGF) expression than do non-TNBC patients [3]. VEGF, a crucial inducer of tumor angiogenesis and vascular permeability, operates through the VEGFR2 receptor tyrosine kinase. Upon VEGF-VEGFR2 binding, VEGFR2 undergoes auto-transphosphorylation, leading to its activation of downstream effectors that promote cancer stem cell (CSC) formation and tumor proliferation, migration, and survival [4]. TNBC is more sensitive to antiangiogenic treatment than is non-TNBC; thus, antiangiogenic agents are an option for treating TNBC [5]. A monoclonal antibody against VEGF (Avastin, bevacizumab) has shown clinical effects in the treatment of TNBC patients, but high dose Avastin can cause serious side effects [6]. The continuation of Avastin treatment until disease progression or the appearance of unacceptable toxicity is still recommended. Blockade of the VEGF/VEGFR2 axis in tumors is expected to inhibit angiogenesis, invasion, and metastasis [7]. However, antiangiogenic drugs have been found to induce distant metastasis by activating hypoxia-induced factor (HIF-1α) and contribute to unfavorable prognosis [8]. The consideration of dose reduction due to high dose Avastin-related adverse effects is important. Additionally, novel strategies of anticancer agents in combination with non-anticancer drugs, that may be more effective for treating TNBC and increasing life expectancy, are suggested [9].

There is a growing interest in investigating certain nutritional supplements to improve anticancer treatments [10]. Studies in recent years show that marine-based fish oil (FO) containing omega-3 fatty acids such as eicosapentaenoic acid (EPA, 20:5n−3) and docosahexaenoic acid (DHA, 22:6n−3), and micronutrient selenium (Se) play pivotal roles in certain cancer treatment of experimental models. Omega-6 fatty acids are reported to stimulate the invasion and metastasis of human breast cancer cell lines in nude mice, whereas EPA and DHA exert their inhibitory effects by suppressing oncogenic growth signaling [11]. EPA and DHA have been shown to reduce VEGF levels and inhibit the expression of epidermal growth factor receptor (EGFR) in tumor lipid raft microdomains and membrane phospholipids, as well as promote tumor apoptosis and block migration and metastasis [12].

Treatment with FO enhanced the antitumor activity of Se in an in vitro preclinical model of lung adenocarcinoma [10]. Patients with breast cancer have lower levels of blood Se than do healthy controls, with the lowest Se levels observed in those patients with stage IV breast cancer [13]. Se induces oxidative stress in tumor tissues and exerts antitumor activity, including the inhibition of tumor growth, VEGF expression, and metastasis; increases the immune response; and decreases antitumor drug resistance [14]. Together, treatments combining anticancer agents with FO and Se may be more effective than the use of anticancer agents.

Nutritional mixtures containing FO and Se may increase the anticancer effects of antitumor agents through several anticancer molecular mechanisms [15]. This study aims at further determining the dose-dependent efficacy of combination treatment using FO and Se with low dose Avastin for improving antitumor effects and the mechanisms of anticancer activity in mice bearing triple-negative 4T1 mammary-carcinoma. The responses to their anticancer treatment via a variety of mechanisms including proangiogenic (growth) factors and their membrane receptors, cytoplasmic signaling pathways (membrane proximal and downstream signaling intermediate), nuclear cyclin and cyclin-dependent kinases, epithelial-to-mesenchymal transition (EMT), and apoptosis were also evaluated.

## 2. Results

### 2.1. Body Weight, Tumor Weight, and Subcutaneous Tumor Size

**Figure 1:**
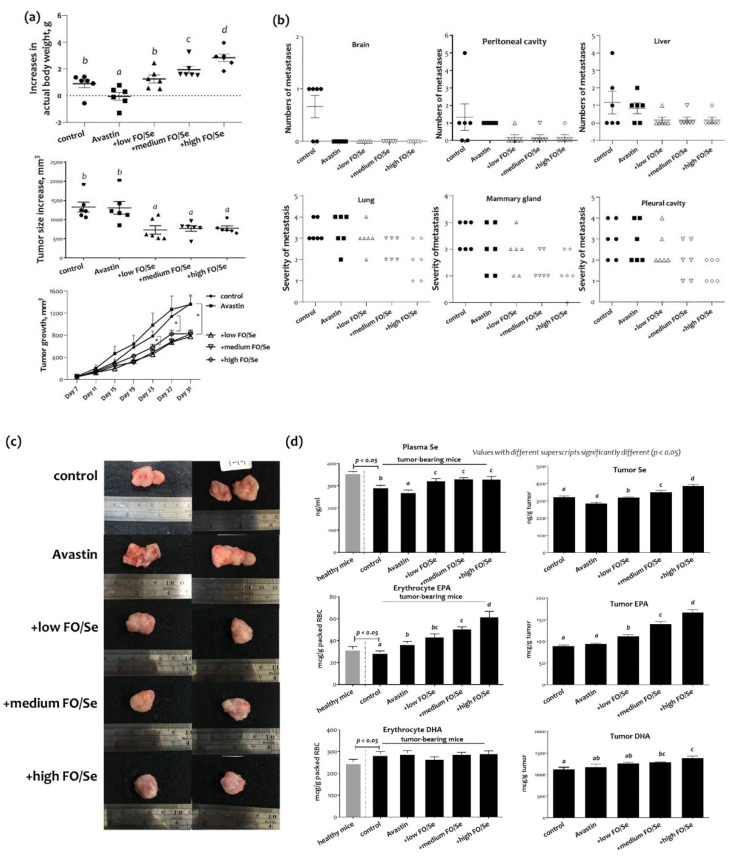
Body weight, tumor growth and metastasis, and levels of eicosapentaenoic acid (EPA), docosahexaenoic acid (DHA) and micronutrient selenium (Se). (**a**) Increases in actual body weight, tumor size, and tumor growth from day 7 to day 31 during experimental period, (**b**) distal tumor metastasis, (**c**) exterior of tumors, and (**d**) Se and EPA/DHA levels in blood and tumor tissues of 4T1 tumor-bearing mice. Values are expressed as mean ± SEM. Values with different superscripts (*a, b, c,* and *d*) significantly different (*p* < 0.05). 4T1 tumor cells (1 × 10^5^) were injected into the subcutaneous region of the mouse right hind thigh at day 0 of the experiment. Tumor-bearing mice at day 7 were randomized into five weight-matched groups of six mice each as follows: (1) control group injected with saline; (2) Avastin group injected intraperitoneally with 5 mg/kg of the Avastin (once every four days); (3) + low FO/Se, +medium FO/Se, and +high FO/Se groups injected intraperitoneally with 5 mg/kg of the Avastin (once every four days) and supplemented with low, medium, and high concentrations of fish oil/selenium supplements by oral gavage twice a day from day 7 to day 31, respectively. The metastasis for lung was graded as 0/no tumor node; 1: 1–15 tumor nodes; 2: 16–30 nodes; 3: 31–35 nodes; 4: 45–60 nodes. Metastasis for mammary gland was graded as 0: no tumor node; 1: 1–4 nodes; 2: 5–8 nodes; 3: 9–12 nodes; 4: more than 13 nodes. Metastasis for pleural cavity was graded as 1: one to two nodes; 2: three to four nodes; 3: five to six nodes; 4: more than seven tumor nodes. Se = selenium; EPA/DHA = eicosapentaenoic acid/docosahexaenoic acid; FO = fish oil. Increases in actual body weight = (changes in body weight from day 7 to 31)–tumor weight.

Increases in actual body weight, subtracting that of the tumor, were higher in tumor-bearing mice treated with Avastin plus marine-based FO and Se (FO/Se) than in those receiving saline or Avastin alone (*p* < 0.05, Figure 1a). Mice in the Avastin group had smallest body weight gains during the experimental period. There were no significant differences in the weights of tumor masses among all groups (data not shown). Mice receiving the antiangiogenic agent Avastin together with FO and Se had significantly smaller tumors than did those treated with Avastin alone (Figure 1a). No significant difference in survival was observed between the five groups, with a survival rate of 100% in all groups.

### 2.2. Distant Metastatic Profiles

Distant metastases in pleural cavity, peritoneal cavity, brain, lung, liver, and mammary gland are shown in Figure 1b. Combination treatment of Avastin and FO/Se showed nonsignificantly lower metastatic potentials in liver, and peritoneum than those in control and Avastin mice. For pleural cavity, severe metastasis (grades 3 and 4) was seen in 66% and 50% of controls and Avastin group, respectively; whereas nonsignificantly lower proportions (34%, 33%, and 0%) were observed in mice cotreatment with Avastin and low, medium, or high levels of FO/Se, respectively. Further, the proportions in grades 3 and 4 of mammary gland metastasis in Avastin/medium or high FO/Se groups (33% and 33%), as well as those in control and Avastin groups (100% and 67%) were seen. Likewise, mice in Avastin with medium or high FO/Se groups have nonsignificantly lower proportions (50% and 33%) in lung metastasis (grades 3 and 4) than those in control and Avastin mice (100% and 83%).

### 2.3. Tumor and Plasma Concentrations of Se and EPA/DHA

Compared with the healthy mice, tumor-bearing mice in the control group had lower plasma levels of Se (Figure 1c). Mice treated with Avastin alone had significantly lower concentrations of Se in plasma and tumors than did mice given only saline in control group. Compared with those treated with Avastin alone, the plasma Se levels in mice treated with FO and Se supplements were higher; the tumor levels of Se in mice treated with FO and Se supplements were dose-dependently higher than those treated with Avastin alone.

As shown in Figure 1c, tumor-bearing control mice had lower erythrocyte membrane EPA and higher DHA contents than did mice in the healthy control group. There were significantly higher contents of EPA but not DHA in mice treated with Avastin as compared with that in mice given saline in control group. Mice treated with low, medium, or high concentrations of the supplements had a higher EPA content in erythrocyte membrane than did those treated with Avastin alone. In contrast, no significant difference in DHA contents of the erythrocyte membrane was observed among all treatment groups that received Avastin with and without FO/Se. There were no significant differences in contents of tumor EPA and DHA in the Avastin group compared with that in control group. Mice treated with Avastin and FO/Se had higher concentrations of EPA and DHA in tumors than did those given only Avastin.

### 2.4. Protein Levels of Tumor Selenoproteins

**Figure 2:**
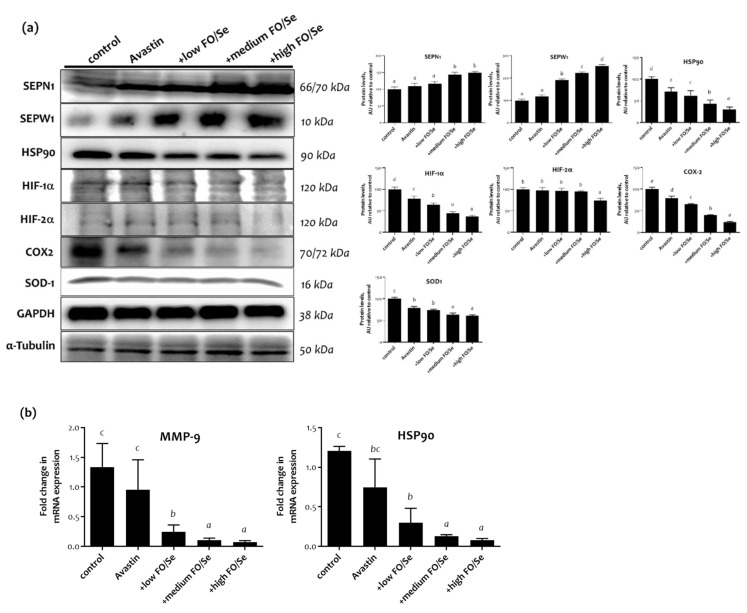
Tumor selenoproteins and HSP90-HIFs-SOD-1-COX-2-MMP9 expression. (**a**) Protein expressions of tumor selenoproteins (SEPN1 and SEPW1), HSP90, HIF-1α/HIF-2α, SOD-1, COX-2, and MMP-9, and (**b**) the levels of relative HSP90 and MMP9 mRNA in tumor tissues of 4T1 tumor-bearing mice were determined by qRT-PCR. Values are expressed as relative reading (mean ± SEM) from at least three or four independent observations. Bars with different superscripts (*a, b, c,* and *d*) are significantly different (*p* < 0.05). Details of groups illustrated are as Figure 1. SEPN1/SEPW1 = selenoprotein N/W; HSP90 = heat shock protein 90; HIFs = hypoxia-inducible factors (HIF-1α and HIF-2α); SOD-1 = superoxide dismutase-1; COX-2 = cyclo-oxygenase-2; MMP9 = matrix metallopeptidase 9.

The expression levels of the selenoproteins SEPN1 and SEPW1 of tumor tissues in the Avastin group were like those in the control group. The levels of SEPW1 in tumor tissues were higher in mice treated with low, medium, or high concentrations of FO/Se supplements than in those treated with Avastin alone (Figure 2a). In addition, higher levels of SPEN1 were observed in Avastin-treated mice that received FO/Se supplements, at medium and high dose.

### 2.5. HSP90/HIFs/COX-2/SOD-1 and MMP-9 in Tumors

Lower expression levels of HSP90 and HIF-1α were observed in mice treated with Avastin alone than in controls, with further lower levels seen in those treated with Avastin plus FO/Se, at all concentrations tested (Figure 2a). Additionally, the lowest level of HIF-2α expression was observed in mice treated with Avastin plus the high concentration of FO/Se supplements. Similar results were obtained when HSP90 mRNA was compared (Figure 2b).

Lower cyclooxygenase (COX)-2 expression was observed in Avastin-treated mice than in controls, with further lower expression levels seen in those treated with Avastin plus the FO/Se supplements, at all concentrations tested (Figure 2a). Additionally, mice treated with Avastin alone exhibited lower expression of antioxidant superoxide dismutase (SOD-1) than did control mice. Avastin-treated mice with the supplements, at medium and high levels, have lower expression of SOD-1 in tumor tissues than did Avastin alone. The matrix metalloprotease-9 (MMP-9) in tumor tissues was significantly lower in mice treated with Avastin alone than in controls and were further lower in those treated with Avastin plus the FO/Se supplements. The MMP-9 mRNA levels in mice receiving combination treatment with Avastin and FO/Se were also lower than in Avastin-alone-treated mice (Figure 2b).

### 2.6. Pro-Angiogenic (Growth) Factors and Their Receptors

#### 2.6.1. VEGF(R)/EGF(R)/FGF(R)/PDGF(R)/TGFβ(R)

**Figure 3:**
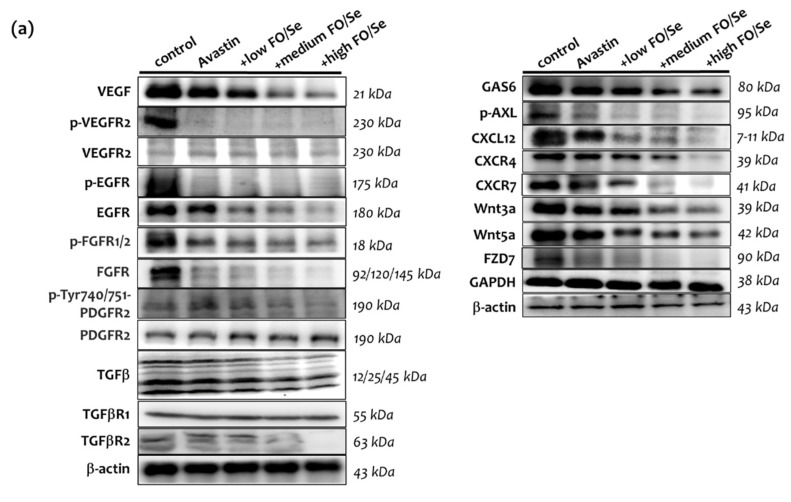

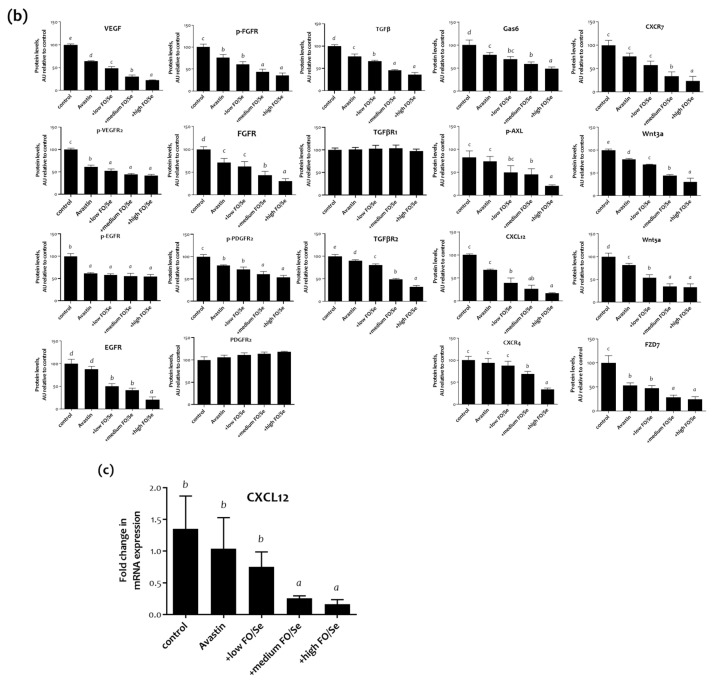
Expression of tumor proangiogenic (growth) factors and their receptors. (**a**) Protein levels of proangiogenic (growth) factors and their receptors, (**b**) densitometric analysis, and (**c**) relative CXCL12 mRNA in tumor tissues of 4T1 tumor-bearing mice were determined by qRT-PCR. Values are expressed as relative reading (mean ± SEM) from at least three or four independent observations. Bars with different superscripts (*a, b, c,* and *d*) are significantly different (*p* < 0.05). Details of groups illustrated are as Figure 1. VEGF = vascular endothelial growth factor; EGF=epidermal growth factor; FGF = fibroblast growth factors; PDGF = platelet-derived growth factor; TGFβ = Transforming growth factor beta; GAS6/AXL = growth arrest-specific 6/receptor tyrosine kinase Axl; CXCL12/CXCR4,7 = stromal cell-derived factor-1/C-X-C chemokine receptor type 4,-7; Wnt/FZD7 = WNT/Frizzled-7.

Mice treated with Avastin alone had lower levels of VEGF and phospho-VEGFR2 in tumors than did saline controls (Figure 3a). Avastin-treated mice receiving FO/Se supplements had much lower levels of VEGF and phospho-VEGFR2 than did those treated with Avastin alone. As seen in Figure 3a, Avastin-treated mice with and without the supplements exhibited significantly lower levels of epidermal growth factor receptor (EGFR) phosphorylation in tumor tissues than did controls. Mice treated with Avastin alone exhibited lower phosphorylation levels of fibroblast growth factor (FGFR) and platelet-derived growth factor receptor (PDGFR2) in tumor tissues than did controls. The level of FGFR and PDGFR2 phosphorylation was higher in mice treated with Avastin alone than in those receiving Avastin plus the supplements, with the phosphorylation levels decreasing with increasing concentrations of FO/Se. For transforming growth factor beta (TGFβ) and TGFβ receptor (TGFβR) expression, mice receiving Avastin alone or Avastin with FO/Se had lower levels of TGFβ and TGFβR2 in tumor tissues. Combined treatment with Avastin and the FO/Se supplements, at all concentrations, contributed more lower levels of TGFβ and TGFβR2 than those when only Avastin alone was used. The expression of tumor TGFβR1 did not differ among any of the treatments or controls.

#### 2.6.2. Gas6/AXL Axis

Mice treated with Avastin alone showed lower expression of growth arrest-specific 6 (Gas6) than did control mice (Figure 3a) and mice treated with Avastin plus supplements at any concentration expressed significantly lower levels of Gas6 than did those treated with Avastin alone. No significant difference was observed in the phosphorylation level of Axl receptor tyrosine kinase (AXL) between Avastin-treated mice and controls. Compared with mice treated with Avastin alone, those receiving Avastin plus the supplements, at all concentrations, exhibited lower levels of AXL phosphorylation.

#### 2.6.3. Chemokine CXCL12/CXCR4, -7 Axis

Higher chemokine CXCL12 (stromal cell-derived factor-1, SDF-1) protein levels were observed in control mice compared with those of treated with Avastin alone or in combination with the supplements, with expression levels decreasing with increasing FO/Se concentrations (Figure 3a). Similar results were obtained comparing C XCL12 mRNA levels (Figure 3b). No significant differences in chemokine receptor CXCR4 levels were observed between control and Avastin-treated mice, without supplements. Mice receiving the FO/Se supplements exhibited lower expression levels of CXCR4 and CXCR7, with expression decreasing in a dose-dependent manner.

#### 2.6.4. Wnt3a, 5a/FZD7 Axis

All Avastin-treated mice exhibited lower expression levels of the canonical WNT ligands Wnt3a and Wnt5a (Figure 3a), with lower levels observed in those treated with supplements than without supplements. Treatment with Avastin alone decreased the expression level of frizzled homolog 7 (FZD7), with such expression lowered further by combined treatment with Avastin and the FO/Se supplements, at medium and high concentrations.

### 2.7. Cytoplasmic Signaling Pathways

#### 2.7.1. PI3K-PTEN-AKT-TSC1/TSC2-mTOR Axis

**Figure 4:**
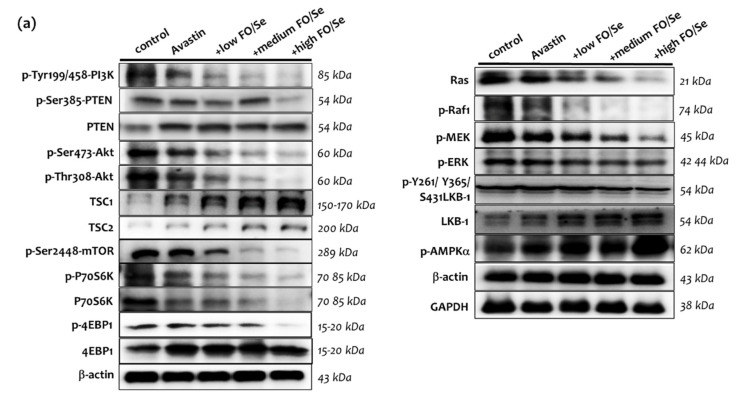

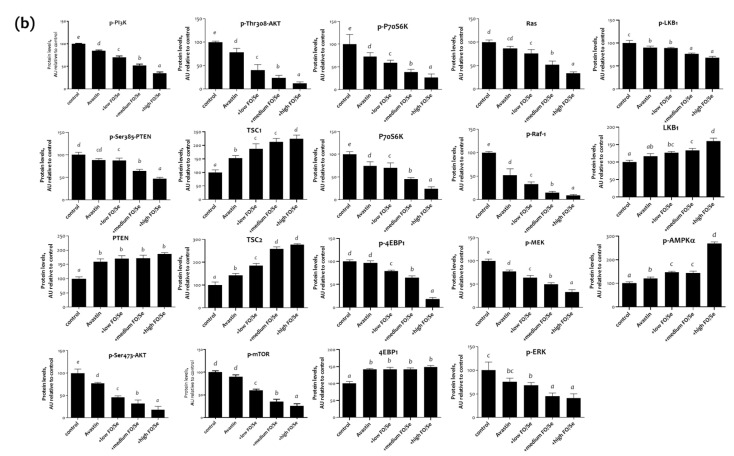
Combination treatment affects tumor PI3K-Ras-LKB1-AMPK signaling pathways. (**a**) Protein levels of PI3K-Ras and LKB1-AMPK pathways, and (**b**) densitometric analysis. Values are expressed as relative reading (mean ± SEM) from at least three or four independent observations. Bars with different superscripts (*a, b, c,* and *d*) are significantly different (*p* < 0.05). Details of groups illustrated are as Figure 1. PI3K = phosphoinositide 3-kinases; PTEN = phosphatase and tensin homolog, a multifunctional tumor suppressor; TSC = tuberous sclerosis complex; mTOR = mechanistic target of rapamycin. PI3K = phosphoinositide 3-kinases; PTEN = phosphatase and tensin homolog; AKT = protein kinase B; TSC = tuberous sclerosis complex; mTOR = mammalian target of rapamycin; MEK = MAPK/ERK kinase; ERK = extracellular signal-regulated kinases; LKB1 = liver kinase B1; AMPK = AMP-activated protein kinase.

Mice treated with Avastin alone exhibited lower phosphorylation of phosphati-dylinositol-3-kinase (PI3K), phosphatase and tensin homolog (PTEN), Ser473-Thr308-Akt, and mammalian target of rapamycin (mTOR), along with higher expression levels of PTEN and tuberous sclerosis proteins 1 and 2 (TSC1 and TSC2) when compared with controls (Figure 4). Combination treatment with Avastin and FO/Se supplements further decreased the phosphorylation levels of PI3K, PTEN, AKT, and mTOR and increased the expression levels of TSC1 and TSC2.

Significantly lower phosphorylation levels of serine/threonine kinase ribosomal protein S6 kinase beta-1 (phospho-p70S6K) were observed in mice treated with Avastin alone than in control mice, with further decreases in phosphorylation seen in with combination treatment. Treatment with Avastin and FO/Se supplements exhibited lower phosphorylation levels of eukaryotic translation initiation factor 4E binding protein 1 (phospho-4EBP1) than in those of treated with Avastin alone.

#### 2.7.2. Ras-Raf-MEK-ERK, and LKB1-AMPK Pathway

Mice treated with Avastin alone exhibited lower levels of Ras, phospho-Raf1, phospho-MEK, and phospho-ERK than did controls (Figure 4). Combined treatment with Avastin and FO/Se supplements further decreased the levels of Ras, phospho-Raf1, phospho-MEK, and phospho-ERK1/2 below those treated with Avastin alone, in a dose-dependent manner. Mice receiving Avastin alone exhibited nonsignificant higher levels of liver kinase B1 (LKB-1) than did controls; addition of the FO/Se supplements, at all concentrations tested, to Avastin treatment significantly increased the levels of LKB-1 as compared to those treated with Avastin alone and controls. Mice treated with Avastin alone exhibited higher levels of phospho-AMPK than did controls, and combined treatment with Avastin and the supplements, at all concentrations tested, significantly increased tumor phosphor-AMPK expression over those treated with Avastin alone.

#### 2.7.3. TGFβ-Smad Pathway

**Figure 5:**
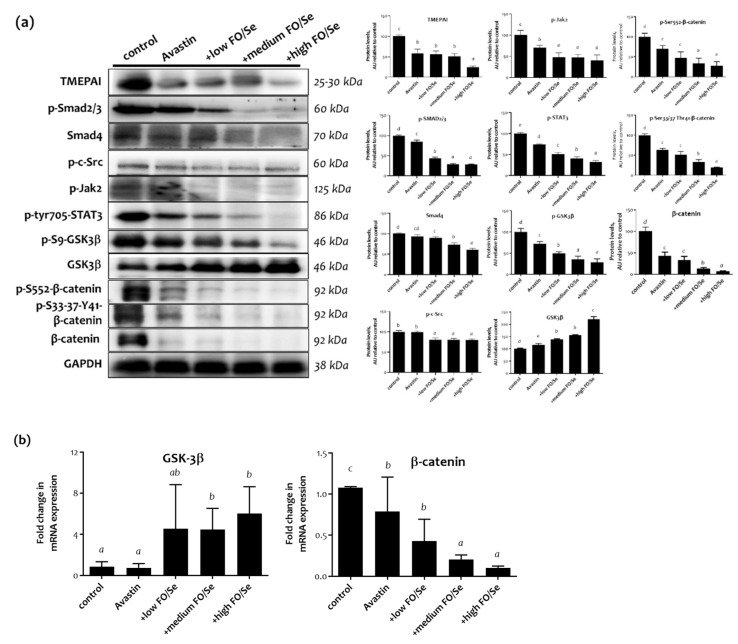
Expression of c-Src-Jak2-STAT3-TMEPAI-Smad and GSK3 β/β-catenin signaling. (**a**) Protein levels of c-Src-Jak2-STAT3-TMEPAI-Smad and GSK3β/β-catenin, and (**b**) relative GSK3β and β-catenin mRNA in tumor tissues of 4T1 tumor-bearing mice were determined by qRT-PCR. Values are expressed as relative reading (mean ± SEM) from at least three or four independent observations. Bars with different superscripts (*a, b, c,* and *d*) are significantly different (*p* < 0.05). Details of groups illustrated are as Figure 1. TMEPAI = prostate transmembrane protein androgen induced 1; GSK3β = glycogen synthase kinase 3β.

Compared with controls, mice treated with Avastin alone exhibited lower levels of phospho-Smad2/3 but not Smad4 (Figure 5). Mice receiving combination treatment of Avastin plus the supplements, at all concentrations tested, had significantly lower levels of phospho-Smad2/3 and Smad4 than did those treated with Avastin alone and controls. Mice treated with Avastin alone had lower levels of transmembrane prostate androgen-induced RNA (TMEPAI) than did controls. Mice received supplements at high concentrations tested have advanced lower levels of TMEPAI.

#### 2.7.4. c-Src-JAK2-STAT3, and GSK3β-β-Catenin Signaling Pathway

Avastin-treated mice exhibited lower levels of phopsho-JAK2 than did controls (Figure 5), with significantly lower levels of phospho-c-Src and phopsho-JAK2 observed in mice treated with Avastin plus the FO/Se supplements. In addition, significantly lower levels of STAT3 phosphorylation were observed in mice receiving the combination treatment, at all concentrations of supplements tested.

Lower protein levels of phospho-GSK-3β and β-catenin were observed in mice treated with Avastin alone than in controls, with further decreases in the levels of phospho-GSK-3β and β-catenin in exhibited in mice treated with Avastin plus FO/Se supplements, at all concentrations tested. At all supplement concentrations, mice receiving the combination treatment exhibited the highest levels of GSK-3β and lowest levels of p-S552-β-catenin and p-S33-37-Y41-β-catenin in tumor tissues. Additionally, higher expression levels of mRNA on GSK3β and lower β-catenin were observed in mice treated with the combination therapy as compared with those in Avastin-alone group.

### 2.8. EMT-Activated Transcription Factors, and Nuclear Cyclin/Cyclin-Dependent Kinases

**Figure 6:**
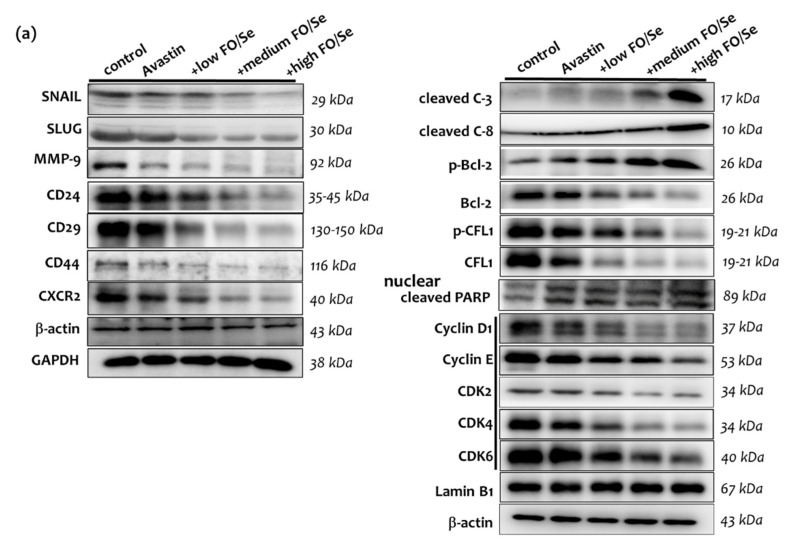

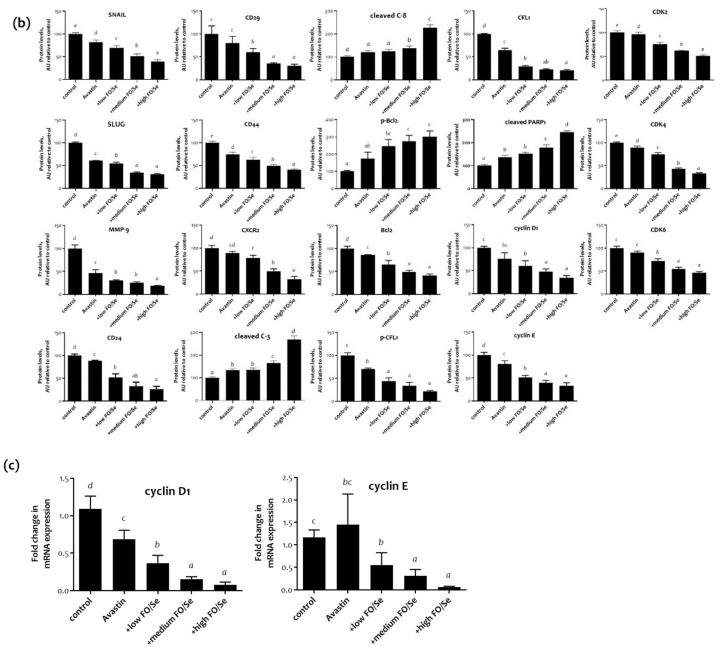
Analysis of epithelial-to-mesenchymal transition, cancer stem cells, cyclins/CDK, and apoptosis in tumor of 4T1 tumor-bearing mice. (**a**) epithelial-to-mesenchymal transition (EMT), cancer stem cells, and apoptosis, (**b**) densitometric analysis, and (**c**) the levels of relative Cyclin D1 and Cyclin E mRNA in tumor of 4T1 tumor-bearing mice were determined by qRT-PCR. Values are expressed as relative reading (mean ± SEM) from at least three or four independent observations. Bars with different superscripts (*a, b, c,* and *d*) are significantly different (*p* < 0.05). Details of groups illustrated are as Figure 1. Epithelial-to-mesenchymal transition transcription factors (SNAIL, SLUG); Cancer stem cell markers-CD24, CD29, CD44, and CXCR2; PARP = Poly(ADP-ribose) polymerase; Cleaved C3 = cleaved caspase-3/Cleaved C8 = cleaved caspase-8; CFL-1 = cofilin-1; CDK = cyclin-dependent kinases.

The levels of epithelial-to-mesenchymal transition (EMT)-activated transcription factors SNAIL and SLUG in tumor tissues were significantly lower in mice treated with Avastin alone than in controls (Figure 6a) and were even further lowered in those treated with Avastin plus the FO/Se supplements. The expression of cyclin E, and cyclin-dependent kinase (CDK)-4 was lower in mice treated with Avastin alone while significantly lower expression of cyclin D1 and E, and CDK-2, -4 and -6 proteins in mice treated with Avastin plus the FO/Se supplements, at all concentrations tested, than in control mice. Additionally, quantitative RT-PCR analysis showed that the mRNA levels of cyclin D1 and E in mice receiving combination treatment were significantly lower when compared with those in Avastin-alone-treated mice (Figure 6c).

### 2.9. Tumor PARP-1, Caspases, Bcl-2, and CFL-1

Poly (ADP-ribose) polymerase (PARP-1), a nuclear protein, is one of caspase substrates. Thus, cleavage of PARP-1 by caspases is regarded as a biomarker of apoptosis. Mice treated with Avastin alone expressed higher levels of cleaved-caspase-3, phospho-Bcl-2, and cleaved-PARP-1 than did controls (Figure 6a). Compared with mice treated with Avastin alone, those receiving Avastin plus medium or high concentrations of the FO/Se supplements expressed significantly higher levels of cleaved-caspase-3, -8 and phospho-Bcl-2, and cleaved-PARP-1. Lower levels of cofilin-1 (CFL-1) phosphorylation were observed in mice treated with Avastin plus FO/Se supplements, at all concentrations tested, than in those treated with Avastin alone.

### 2.10. CSCs Markers

The expression levels of CD24, CD29, CD44, and chemokine receptor CXCR2, potential CSC markers in TNBC, were lower in Avastin-treated mice than in controls (Figure 6a), with lower expression in mice treated with Avastin plus the FO/Se supplements than with Avastin alone.

## 3. Discussion

**Figure 7:**
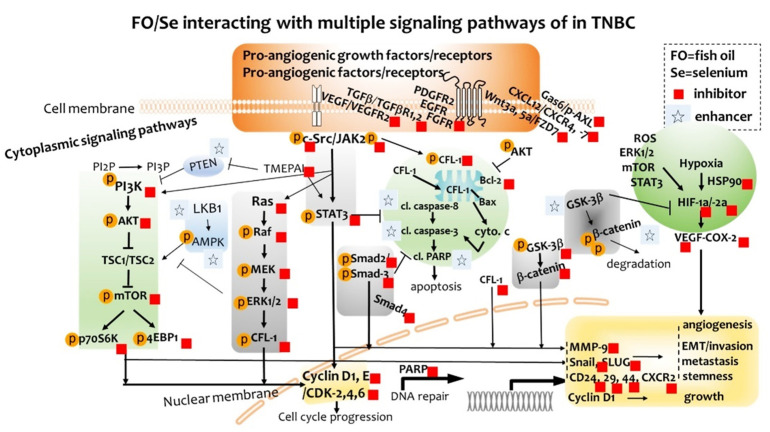
Schematic diagram showing combination treatment with FO and Se increases the therapeutic efficacy of Avastin against TNBC through multiple signaling pathways in the membrane, cytoplasmic, and nucleic targets.

Anticancer agent Avastin is still commonly used for the treatment of TNBC, although investigating the anticancer effects of Avastin was not the primary focus of this work. The present study evaluated the dose-dependent efficacy and associated anticancer mechanisms of combination treatment with the antiangiogenic agent Avastin plus marine-based FO and Se supplements in TNBC-bearing mice. We observed that in low dose Avastin-treated mice, dietary supplementation with FO and Se markedly decreased the tumor size, inhibited EMT and metastasis, reduced the CSCs, and increased apoptosis in a dose-dependent manner. Furthermore, we observed that the anticancer mechanisms underlying this combined treatment involve the inhibition of multiple proangiogenic (growth) factors and their receptors and the targeting of cytoplasmic and nuclear signaling pathways, as shown in Figure 7.

### 3.1. EPA/DHA Levels in TNBC

Throughout the experimental period, no deaths were observed, as results presented here show. A longer period of treatment is required in order to compare cancer survival rates, although cancer survival was not the primary purpose of this investigation. The anticancer mechanisms of EPA and DHA have been shown to readily incorporate into tumor lipid and phospholipid, thus altering structural integrity; additionally, the bioactive lipid metabolites derived from EPA and DHA are involved in anticancer activity [12]. EPA is incorporated into tumors to a greater extent than DHA in MDA-MB-231 human breast cancer cells [16]. Treatment of Avastin-treated mice with FO/Se show significantly increased levels of EPA in tumor tissue. The levels of DHA in tumors, by contrast, are clearly increased only in the high FO/Se group, compared with Avastin group. Thus, EPA may have greater anticancer effects than DHA in Avastin-treated tumor-bearing mice. A previous study suggests that DHA is a more powerful antineoplastic agent, whereas EPA and DHA may act either through the same or different antitumor mechanisms of various tumor types [17].

### 3.2. Se Accumulation and Selenoprotein Expression in TNBC

A search of the literature does not find any documentation of a drop in the essential micronutrient Se in tumors and plasma under Avastin treatment, whereas this phenomenon may be attributed to anti-angiogenesis treatment and increased excretion of Se which is avastin-induced proteinuria. Se is incorporated as selenocysteine (Sec) into selenoproteins; thus, selenoproteins are the primary effector molecules of Se. The regulation of selenoprotein expression is important for intracellular Se homeostasis, redox signaling, immune responses, and modulation of the cell cycle [18]. However, the anticancer role of selenoproteins remains to be elucidated. A recent study has shown that SEPW1 is required for normal cell cycle progression and EGF-induced EGFR activation [19]. Our present results show that Se supplementation, in a dose-dependent manner, induced greater expression of tumor SEPN1 and SEPW1 and increased Se accumulation in Avastin-treated tumor-bearing mice. Tumor Se accumulation has been proposed to trigger pro-oxidative apoptosis and inhibit VEGF expression [14,18]. Thus, the pro-oxidant property of Se and upregulation of SEPN1/SEPW1 by Se accumulation in TNBC tumors may play crucial roles in the modulation of anticancer mechanisms.

### 3.3. FO/Se and Pro-Angiogenic Growth Factors in TNBC

The expression levels of the proangiogenic growth factors VEGF, EGF, FGF, and PDGF and their corresponding receptors are significantly higher in TNBC than in non-TNBC [20]. These growth factors bind with high affinity to their cognate tyrosine kinase receptors (VEGFR, EGFR, FGFR, and PDGFR), which play pivotal roles in angiogenesis, tumor growth, and survival, and are associated with poor prognosis, high metastasis, and lower overall survival and disease-free survival [9,20]. Additionally, TGF-β is a multifunctional cytokine that binds to its serine/threonine kinase receptor (TGF-βR), playing a vital role in the CSC population, anticancer drug resistance, and the processes of EMT in TNBC [21].

EPA/DHA is reported to decrease the levels of VEGF and phosphorylated EGFR in MDA-MB-231 breast cancer cells and inhibit microvascular formation in tumor-bearing mice [22]. Advanced breast cancer patients who received chemotherapy plus FO had lower VEGF levels than did patients treated with chemotherapy alone [23]. Furthermore, Se treatment of human breast cancer cells and ovarian cancer cells has been shown to inhibit VEGF and MMP expression [14,24]. TNBC tumor-bearing mice receiving FO/Se had significantly lower tumor VEGF protein expression [15]. Our present results show that treatment with low-dose Avastin combined with FO and Se exerts dose-dependent inhibitory effects on the activation of multiple proangiogenic growth factors/receptors in TNBC tumors. Avastin, a VEGF inhibitor, in combination with FO and Se supplements can be regarded as a multikinase inhibitor targeting different receptors tyrosine kinase.

### 3.4. FO/Se and Pro-Angiogenic Factors in TNBC

Furthermore, Gas6 is the activating ligand of the AXL oncogenic receptor tyrosine kinase. The binding of Gas6 to AXL induces pathways involved in tumor cell growth, invasion, EMT, angiogenesis, drug resistance, immune regulation, and CSC maintenance. Treatment with anti-AXL antibody inhibits AXL-dependent EMT and tumor growth in TNBC patients [25]. Binding of the chemokine CXCL12 to its receptors CXCR4 and CXCR7 is positively associated with the progression of breast cancer; high expression of both CXCR4/CXCR7 has been implicated in the activation of EMT, tumor stemness, and chemoresistance [26]. Moreover, the CXCL12 produced by tumor cells attracts CXCR4+ Treg and pro-tumorigenic myeloid-derived suppressor cells, further promoting tumor progression [27]. Frizzled receptors (FZDs) are a family of transmembrane receptors for Wingless-type (Wnt) ligands in the Wnt pathway. FZD7 expression is significantly higher in TNBC than in non-TNBC tumor tissues. Furthermore, upregulation of FZDs plays a crucial role in tumor angiogenesis, invasion, stemness, and chemoresistance [28]. Thus, inhibition of the Gas6/AXL, CXCR4/CXCR7/XCL12 chemokine axis and Wnt3a, -5a/FZD7 axis is also a potential therapeutic strategy for TNBC.

Few studies have investigated the effects of EPA/DHA or Se on the Gas6/AXL or CXCL12-CXCR4/CXCR7 axis. Se deficiency increases Wnt expression levels in cardiac tissue [29]; in contrast, Wnt3a expression was observed to reverse DHA-induced growth inhibition and apoptosis in human pancreatic cancer cells [30]. The results of the present study demonstrate that combination treatment with FO and Se may suppress nonclassic proangiogenic factors (and receptors) Gas6/AXL, CXCL12/CXCR4/CXCR7, and Wnt3a/Wnt5a/FZD7 that are essential for angiogenesis and progression of TNBC, in a dose-dependent manner. Thus, the combination of FO with Se is considered a potential mediator for tyrosine kinase and G protein-coupled receptor kinase and cancer stemness.

### 3.5. FO/Se and PI3K-PTEN-AKT-mTOR Signaling in TNBC

Hyperphosphorylation of the oncogenes PI3K, AKT, and mTOR is common in TNBC and has been involved in chemoresistance and survival of TNBC [1]. The tumor suppressor PTEN de-phosphorylates phosphatidylinositol-3,4,5-trisphosphate (PIP3) to PIP2, thereby down-regulating AKT, resulting in blockade of the signaling pathway. AKT activation leads to cell growth by activating mTOR through TSC1/2 phosphorylation, while increased levels of TSC1/TSC2 inhibit the mTOR pathway; mTOR positively regulates 4E-BP1 and p70S6k, which are activated in a variety of cancers. High expression of PI3K, phospho-mTOR, phospho-4E-BP1, and phospho-p70S6k has been implicated in TNBC recurrence, poor overall survival, and poor progression-free survival.

Recent studies have shown that treatment with EPA-, DHA- or Se-containing compounds decreases the phosphorylation of AKT (Ser473, not Thr308) and decreases PI3K activity in cancer cell lines and tumor-bearing mice [12,31,32]. Furthermore, temsirolimus is the only FDA-approved mTOR inhibitor, whereas FO combined with Se here play a mimetic for mTOR inhibitor. The present results demonstrate that combination treatment with FO and Se inactivated AKT by decreasing phosphorylation of both Ser473 and Thr308, and down-regulated PI3K/mTOR/4E-BP1/p70S6k phosphorylation and increased PTEN and TSCA/TSC2 expression in TNBC tumors in a dose-dependent manner. Thus, FO/Se treatment not only restored tumor suppressor PTEN expression, but also exhibited potent inhibition of PI3K/mTOR signaling.

### 3.6. FO/Se and Ras-Raf-MEK-ERK Signaling in TNBC

EGFR activation triggers the Ras-Raf-MEK-ERK cascade in tumor cells. Ras is the upstream effector in the Raf-MEK-ERK signaling pathway. Tumor samples from patients receiving chemotherapy were shown to have elevated levels of Raf-MEK-ERK phosphorylation, which may contribute to resistance and relapse in TNBC [33]. Treatment with an antiangiogenic agent Avastin plus chemotherapy decreased the Ras expression level in patients with advanced NSCLC [34]. Our results show that following combination treatment with Avastin plus FO and Se, Ras-Raf-MEK-ERK signaling was significantly downregulated in TNBC tumor tissues in a dose-dependent manner. Thus, the FO/Se combination may have therapeutic value in treating TNBC via regulation of the Ras-Raf-MEK-ERK signaling.

### 3.7. FO/Se and TGFβ-Smad2/3-TMEPA1 Signaling in TNBC

Activation of TGFβR results in the phosphorylation of Smad2 and Smad3, which then form a heterodimeric complex with Smad4 that induces the transcription of target genes. This pathway is commonly upregulated and associated with tumor angiogenesis, progression, EMT, and chemoresistance in TNBC [35]. A previous study showed that Se treatment of human umbilical vein endothelial cells (HUVECs) attenuated TGF-β activation and phosphorylation of Smad2 and Smad3, and reduced Smad4 expression [36]. TMEPA1 may be a downstream effector of Smad and regulator of PTEN; in Smad3-knockdown TNBC cancer cells, decreased growth, motility, and invasion were reversed by overexpressing TMEPA1. Upregulated TMEPA1 has been shown to decrease PTEN, resulting in increased TGF-β–dependent tumor growth, motility, invasion, and cancer progression in TNBC [37]. Thus, the TGF-β-Smad2/Smad3-TMEPA1-PTEN axis may play a pivotal role in targeted therapy for TNBC. The present results show that the tumor tissues of TNBC mice treated with a combination of low-dose Avastin and FO with Se have significantly lower levels of Smad2/Smad3 phosphorylation, Smad4, and TMEPAI than those of mice treated with Avastin alone.

### 3.8. FO/Se and JAK2-STAT3 Signaling in TNBC

The Janus kinase (JAK)-independent tyrosine phosphorylation of signal transducers and activators of transcription (STAT) likely occurs through regulation of the nonreceptor tyrosine c-Src kinase. Thus, c-Src is a potential target for TNBC treatment. Activation of the JAK2/STAT3 pathway is reported to play critical roles in TNBC, promoting CSC persistence, anti-apoptosis, and radiotherapy resistance [38,39]. Upon binding to their ligands, EGFR and FGFR are also activated, triggering the tyrosine phosphorylation cascade that activates JAK2/STAT3 signaling and the expression of downstream target genes involved in EMT and chemotherapy resistance [39].

The present results demonstrate that combination treatment using Avastin with FO and Se markedly reduced phosphorylated c-Src, JAK2, and STAT3 in a dose-dependent manner compared with that of Avastin alone.

### 3.9. FO/Se and LKB1-AMPK Signaling in TNBC

The LKB1-AMPK cascade is well known to be pivotal in tumor invasion and migration. The tumor suppressor LKB1 encodes a serine/threonine kinase that directly results in AMPK phosphorylation in tumors, suggesting that the tumor suppressor effects of LKB1 may be mediated by AMPK [40]. A recent study shows that AMPK levels are higher in TNBC than in non-TNBC tumor tissues, while decreased levels of phospho-AMPK are positively associated with higher histological grade/axillary node metastasis and anticancer drug resistance in TNBC [41]. Furthermore, phosphorylation of AMPK significantly suppresses cyclin D1, phosphorylation of EGFR and mTOR, and Src-activated STAT3 signaling. The administration of Se is reported to induce AMPK phosphorylation and activate apoptotic caspase-3 in HT-29 colon cancer cells [42]. In non-TNBC MCF-7 cancer cells, DHA treatment increased LKB1 activity, further increasing AMPK phosphorylation and mTOR inhibition [43]. The results of the present study show that compared to Avastin treatment alone, combination treatment with FO and Se upregulated LKB1 and phospho-AMPK in TNBC tumor tissues.

### 3.10. FO/Se and GSK-3β-β-Catenin Signaling in TNBC

GSK-3β is also a serine/threonine kinase that plays a crucial role in the Wnt/β-catenin signaling cascade. Upon Wnt activation, GSK-3β is phosphorylated, followed by the arrest of β-catenin phosphorylation, thereby upregulating the expression of β-catenin protein. β-catenin overexpression is strongly implicated in the chemoresistance of CSCs and increases the risk of lung and brain secondary metastases in TNBC patients [39]. On the other hand, GSK3β activation down-regulates the Wnt/β-catenin signaling pathway, suppressing the EMT, tumor growth, CSC, and antiapoptotic activity in tumor cells [44]. Furthermore, GSK-3β promotes tumor cell apoptosis by inhibiting HIF-1α and facilitating apoptotic transcription factors. Thus, activation of GSK-3β and β-catenin suppression are potential therapeutic targets for TNBC treatment.

Administration of DHA or EPA could cause dephosphorylation of GSK-3β, thus contributing to β-catenin degradation and apoptosis in hepatocellular carcinoma cells [45]. Additionally, Se represses the levels of anticancer agent induced GSK3β phosphorylation and enhancing efficacy of reducing toxicity [46]. The present results show that the combination treatment of Avastin with FO and Se significantly increased the expression of GSK3β and decreased β-catenin in tumor tissues.

### 3.11. FO/Se and Apoptosis in TNBC

Both caspase-3 and caspase-8 are a family of protease enzymes playing vital roles for the apoptotic signaling cascades. Cleaved caspases-3/-8-positive patients have been shown to increase chemotherapy response and apoptosis in TNBC [47]. After induction of apoptosis, cytochrome c is released from mitochondria to the cytosol; whereas overexpression of antiapoptotic Bcl-2 prevents the efflux of cytochrome c from mitochondria. CFL-1 belongs to the actin depolymerization factor/cofilin family involved in chemotaxis and invasion of various tumors. During apoptosis, CFL-1 interaction with apoptosis regulator Bax (bcl-2-like protein 4) followed by CFL-1, translocates from cytosol into the mitochondria, resulting in cytochrome c released from mitochondria. On the other hand, CFL-1 phosphorylation by activated ERK1/2 will be associated with EMT, poor prognosis and worse overall survival in breast cancer [48]. In the present investigation, we observed a greater extent of apoptosis in TNBC mice receiving combined treatment with Avastin and FO/Se than in those treated with Avastin alone.

### 3.12. FO/Se and Cyclin-CDKs in TNBC

Dysregulation of the cyclin and cyclin-dependent kinases (CDKs) are implicated in uncontrolled cancer cell proliferation, growth, and division. Activation of upstream mitogenic PI3K-AKT-mTOR and Ras-Raf-MEK-ERK signaling can enhance cyclin D-dependent CDK4/6 expression. Thus, the inhibitors of cyclins and CDK 2/4/6 have been studied in combination with anticancer agents in breast cancers [49]. The results of the present study show that compared to Avastin treatment alone, combination treatment with FO and Se exerted a greater decrease in nuclear cyclin D1/E and CDK-2,4,-6 in TNBC tumor tissues.

### 3.13. FO/Se and Hypoxia-HIF-COX-2 in TNBC

Tumor hypoxic microenvironment has been shown to provoke the activation of angiogenesis factors such as VEGF/EGF/TGF-β, CSCs and EMT, and drug resistance in a variety of tumor types [18,50]. HIF-1α and HIF-2α are crucial transcription factors responsible for the regulation of hypoxic stimulation. In response to hypoxic conditions, high expressions of HIF-1α and HIF-2α have been correlated with poor prognosis of patient outcome in various tumor forms [50]. Additionally, HSP90 is a crucial chaperone protein, and its over-expression in TNBC tumors is highly correlated with activation of EGFR, EMT, HIF-1α, as well as AKT/MEK/ERK and JAK2/STAT3 signaling, and cell cycle regulatory factors [51]. Activation of either the PI3K-Akt-mTOR or Ras-Raf-MEK-ERK signaling axis was shown to induce COX-2 expression in a variety of tumors; overexpression of COX-2 by HIF-1α induction further increased tumor cell proliferation, angiogenesis, drug resistance, and decreased apoptosis [43,52].

Methylseleninic acid (MSA), an organic Se compound, possesses the potential to reverse chemoresistance, which is attributed to inhibition of HIF-1α by MSA [53]. Se has been shown to activate AMPK, thereby contributing to ERK downregulation, resulting in reduced COX-2 expression in HT-29 colon cells [54]. Our results show that following combination treatment with Avastin plus FO and Se, exerts dose-dependent inhibitory effects on the activation of HIF-1α/HIF-2α, HSP90 along with decreased expression of COX-2 in TNBC tumor tissues in a dose-dependent manner.

### 3.14. FO/Se and Self-Renewal in TNBC

CSCs, known to serve as tumor initiators, can induce the EMT and are highly resistant to conventional chemotherapies. High expression of CD24 and CD29 breast CSCs has been observed in TNBC and may contribute to chemotherapy resistance [55]. CXCR2 is considered a novel CSC marker for only TNBC and is also involved in breast cancer angiogenesis and metastasis, treatment resistance, and recurrence [56]. The results of the present study demonstrate that combination treatment with FO and Se inhibit CSCs that are essential for angiogenesis and progression of TNBC, in a dose-dependent manner. Thus, FO/Se can play an important role in the inhibition of TNBC-associated stemness.

### 3.15. FO/Se and EMT in TNBC

The EMT is a critical cellular program that enables nonmotile epithelial cells to become invasive mesenchymal cells; thus, the EMT plays a major role in the metastasis of TNBC [57]. The EMT process has also been implicated in chemoresistance, tumor recurrence, induction of CSC properties, and degradation of the extracellular matrix. Proteins known to be potent inducers of the EMT include the transcription factors SNAIL and SLUG and the signaling molecules TGFβ and Wnt [21]. In addition, matrix metalloproteases (MMPs) have been well recognized as promoting vascularization, degrading the extracellular matrix, and increasing EMT, thus facilitating tumor invasion [58]. MMP9 expression is regulated by EGF, FGF, and HSP90, as well as by oncogenic proteins including Ras, c-Src, and STAT3; additionally, high levels of MMP-9 expression in tumors correlate negatively with disease-free survival and progression-free survival in TNBC [59].

Our present results show that treatment with low-dose Avastin combined with FO and Se exerts dose-dependent inhibitory effects on the activation of the EMT as well as the induction of MMP-9 expression in TNBC tumors. Avastin in combination with FO and Se supplements can be regarded as an inhibitor targeting EMT.

## 4. Materials and Methods

### 4.1. Anti-Angiogenic Agent and Nutritional Supplements

The anti-VEGF agent Avastin (Roche Diagnostics GmbH, Mannheim, Germany) was purchased from commercial sources. As described previously [15], a control-based powder that was free from FO and Se (127.5 mg carbohydrate, 30.0 mg fat, and 65.5 mg protein per Kcal) was purchased from Do Well Laboratories, Inc. (Irvine, CA, USA). The Supplemental marine-based FO and Se yeast (Do Well Laboratories) were mixed with the control powder because these components are generally insoluble in water under normal conditions. Thus, the final concentrations of EPA, DHA, and elemental Se in low, medium, and high doses of FO/Se supplements were 5.1 mg, 3.7 mg, and 2.7 μg/g; 9.1 mg, 6.9 mg, and 4.0 μg; 10.7 mg/g, 8.3 mg, and 6.7 μg/g, respectively.

### 4.2. Antibodies

Mouse MMP-9 (1:1000, #2270), SNAIL (1:1000, #3879), SLUG (1:1000, #9585), caspase-3 (1:1000, #9662), caspase-8 (1:1000, #4790), VEGFR2 (1:1000, #2479), phospho-Tyr1175-VEGFR2 (1:800, #2478), phospho-Tyr1068-EGFR (1:1000, #2234), FGFR (1:1000, #9740), PDGFR2 (1:1000, #3169), phospho-Tyr751-PDGFR2 (1:800, #3166), TGFβ (1:2000, #3711), phospho-Tyr199/458-PI3K (1:1000, #4228), phospho-Ser473-Akt (1:1000, #4058), phospho-Thr308-Akt (1:1000, #9275), phospho-Ser2448-mTOR (1:1000, #5536), PTEN (1:1000, #9552), phospho-Ser385-PTEN (1:1000, #9551), TSC1 (1:1000, #4906), TSC2 (1:1000, #4308), phospho-Thr172-AMPKα (1:1000, #2535), phospho-Tyr416-c-Src (1:1000, #2101), phospho-Tyr1007/1008-Jak2 (1:500, #3776), phospho-Tyr705-STAT3 (1:1000, #9131), phospho-Ser428-LKB-1 (1:1000, #3482), LKB-1 (1:1000, #3047), GSK3β (1:1000, #9832), phospho-Ser9-GSK3β (1:1000, #5558), phospho-Ser552-β-catenin (1:1000, #5651), phospho-Ser33/37-Thr41-β-catenin (1:1000, #9561), p70S6K (1:1000, #9202), phospho-Ser465/467-Ser423/425-Smad2/3 (1:1000, #8828), Smad4 (1:1000, #38454), phospho-Thr421-Ser424-p70S6K (1:1000, #9204), phospho-Ser338-Raf1 (1:1000, #9427), phospho-Ser217/221-MEK (1:1000, #9154), 4EBP-1 (1:1000, #9644), and phospho-Thr37/46-4EBP1 (1:1500, #2855) were purchased from Cell signaling (Beverly, MA). Bcl-2 (1:5000, sc-16323), VEGF (1:1000, sc-7269), HSP90 (1:1000, sc-13119), Cyclin D1 (1:1000, sc-450), Cyclin E (1:1000, sc-247), CDK2,4,6 (1:1500, sc-6248/23896/sc7961), Wnt3a (1:1000, sc136163), CFL1 (1:1000, sc-53934), COX-2 (1:1000, sc-19999), β-catenin (1:1000, sc7963), and phospho-Ser 3-CFL1 (1:1000, sc-271923) were obtained from Santa Cruz Bio-technology (Santa Cruz, California). SEPW1 (1:1000, GTX48717), Gas6 (1:1000, GTX31628), phospho-FGFR1/2 (1:1000, GTX32182), phospho-Thr202-Tyr204-ERK (1:1000, #GTX59568), and Wnt5a (1:1000, GTX111187) were purchased from GeneTex (Hsinchu City, Taiwan). CD24 (1:2000, #2514189), CD44 (1:1000, AB2082), CXCR4 (1:1000, AB1847), EGFR (1:1000, #2193153), GAPDH (1:2000, MAB 374), and β-actin (1:4000, MAB 1501) were obtained from Millipore (EMD Millipore). FZD7 (1:1000, ab64636), CD29 (1:2000, ab179471), HIF-1α (1:500, ab42091), HIF-2α (1:1000, ab109616), TGFβR1 (1:1000, ab235178), TGFβR2 (1:1000, ab61213), CXCR2 (1:1000, ab9938), CXCR7 (1:1000, ab138509), and α-Tubulin (1:2000, ab4074) were provided from Abcam (Cambridge, MA).

Antibodies including CXCL12 (1:1000, #79014, R&D Systems), TMEPAI (1:800, PA5-72873, Thermo Fisher), phospho-Tyr698/Tyr702/ Tyr703-AXL (1:1000, orb4400, Biorbyt), Ras (1:1000, 3233-S, Epitmics), Lamin B1 (1:2000, E-AB-31901, Elabscience), and SEPN1 (1:1000, 55333-1-AP, Proteintech) were also used in the present study.

### 4.3. Cell Culture and Animal Experiment

Mouse 4T1 tumor cell line (CRL-2539) was obtained from the American Type Culture Collection (Rockville, MD, USA), and maintained in RPMI 1640 medium (31800-022; Gibco BRL, Gaithersburg, MD, USA) supplemented with 10% (*v/v*) heat-inactivated fetal bovine serum in a humidified 95% air and atmosphere of 5% CO2 at 37 °C.

Animal experiment were conducted following procedures approved by the Institutional Animal Care Committee of Hung Kuang University. Female mice of the sub-strains BALB/ cByJNarl were obtained from the National Animal Laboratory Breeding Research Centre (Nangang Taipei, Taiwan) at age of seven weeks and maintained on a controlled 12 h light/dark period at 24 ± 1 °C and 60–70% relative humidity. A two-week acclimatization period was allowed for the animals before the experiment, and rodent chow (Ralston Purina, Lab Diet #5001, St. Louis, MO, USA) and distilled deionized water were made available *ad libitum* to all animal throughout the study.

Mouse 4T1 tumor cells (1 × 10^5^) were injected into the subcutaneous region of the mouse right hind thigh at day 0 of the experiment. Tumor-bearing mice at day 7 were randomized into five weight-matched groups of six mice each as follows: Group 1 (control, no treatment with Avastin) was injected saline (0.9% NaCl); Group 2 (Avastin) was injected intraperitoneally with 5 mg/kg of the Avastin (once every four days); Groups 3, 4, and 5 (+ low EPA/DHA/Se, + medium EPA/DHA/Se, and + high EPA/DHA/Se) were injected intraperitoneally with 5 mg/kg of the Avastin (once every four days) and supplemented with 0.4 g of low, medium, and high concentrations of EPA/DHA/Se supplements by oral gavage twice a day from day 7 to day 31, respectively. The volume of tumor was calculated using the formula (X^2^Y)/2 for caliper measurements, where X and Y are the short and long diameters, respectively. Tumor growth-curves calculated by caliper measurements every three days. At the end of the study (day 32), animals were sacrificed after blood collection; primary tumors were also carefully removed and weighed. Additionally, metastatic nodules (degrees) on the tissue surfaces (liver, brain, lung, and mammary gland), pleural cavity, and peritoneal cavity of animals were examined grossly.

### 4.4. Measurement of Omega-3 Fatty Acid EPA and DHA

Briefly, the tumor tissues, erythrocyte membranes, and nutritional supplements were dissolved in a mixture of methanol and benzene (4:1, *v/v*) and then mixed and dried with acetyl chloride twice according to protocols reported previously [16]. The reaction mixture was stirred at 40 °C for 1 h and then heated at 100 °C for 1 h. The mixture was cooled to room temperature, and 3 mL of 6% K_2_CO_3_ and 1 mL of benzene aqueous solutions were mixed thoroughly. The benzene layer was removed and transferred to a gas chromatography vial for analysis. The extract was injected into Agilent technologies 7890A gas–liquid chromatographs fitted with a 100 m × 0.25 nm × 0.2 μm silica capillary column (Agilent J&W 112-88A7; Agilent technologies Inc., Palo Alto, CA, USA) and analyzed using a flame ionization detector (FID).

### 4.5. Determination of Se Content

Atomic absorption spectrophotometry (AAS, 932AA; GBC, Melbourne, Australia) coupled to a hydride generation accessory (HG 3000; GBC) was used to determine the Se concentrations in plasma, tumor tissues, laboratory chow, and nutritional diets according to protocols reported previously [14]. A multielement hollow-cathode lamp (Photron Pty. Ltd., Victoria, Australia) was used as a light source. For Se, the wavelength was 196.0 nm and the spectral bandpass was 0.5 nm. Briefly, the samples were digested in a heated mixture of nitric and perchloric acids (ratio, 2:1) using a multistep process. Selenate was reduced to selenite in the acidified solution in 30 min at a block temperature of 150 °C. The digested solution was then placed in the AAS after selenite was reduced to elemental Se.

### 4.6. Western Blot Analysis

Tumor tissues were extracted in a homogenization buffer containing 1% Nonidet P-40, 0.1% SDS, 0.5% sodium deoxycholic acid, supplemented with a protease inhibitor cocktail (Roche). Nuclear extracts were also prepared by using cell nuclear protein extraction kit (Bio-PNF-20, Biokit Biotech, Inc., Miaoli, Taiwan). Briefly, add buffer A to pieces of tumor tissue and homogenized, and further centrifuged at approximately 860× *g* at 4 °C for two minutes. The resulting supernatants were transferred to new tubes, and centrifuged at 6200× *g* for eight minutes. Remove the supernatant, add 50 μL buffer C to the remaining pellets, and further centrifuged at 16,200× *g* for 12 min, and collected the supernatant. The protein concentrations of lysates were determined by Bio-Rad Protein Assay (Bio-Rad, Hercules, CA, USA) using a series of bovine serum albumin as standards. Approximately 50 μg of the denatured proteins was electrophoretically separated on SDS-poly-acrylamide 6–12% gels and then, transferred onto nitrocellulose membranes and incubated with different primary antibodies, followed by incubation with horseradish peroxidase-conjugated goat antimouse IgG antibody. β-actin, GAPDH, α-tubulin, or lamin B1 was used as loading control probing with β-actin/GAPDH/α-tubulin/lamin B1 rabbit monoclonal. Proteins bound to specific antibodies were then visualized using an enhanced chemiluminescence detection kits (PerkinElmer Life Sciences Inc. Waltham, MA, USA), according to the manufacturer’s protocol. Signal intensities were quantified using the FUJI LAS-4000 system and Multi Gauge 3.0 software (Fuji, Japan).

### 4.7. RNA Extraction and Real-Time PCR Analysis

RNA samples were prepared using the Bio-Rad RNA kit (Bio-Rad Lab, Hercules, CA, USA) according to the established manufacturer’s protocol. The concentration and purity of RNA was determined by measuring the UV absorbance at 260 nm and 280 nm. 5 ng of total RNA was used for cDNA synthesis using a thermocycler (T100 Thermal Cycler) with the iScript cDNA synthesis kit (Bio-Rad Lab, Hercules, CA, USA). Then, mRNA transcription level was assessed with RT-quantitative PCR using the SYBR Green (Bio-Rad Lab, Hercules, CA, USA) in CFX Connect RT-PCR detection system, and the following primers such as MMP-9 (forward, 5′-ACTTGAAGTCTCAGAAGGTGGA-3′; reverse, 5′-GCAGAAATAGGCTTTGTCTTGGT-3′), HSP-90 (forward, 5′-TGAAGCATTAGAGATCAACTCGC-3′; reverse, 5′-GAAATTGCCCAGCTCATGTCC-3′), CXCL12 (forward, 5′-CGGTTCTTCGAGAGCACAT-3’; reverse, 5′-AATGCACACTTGTCTGTGTTGT-3′), GSK3β (forward, 5′-TCTGGAGAACTGGTTGCCATA-3′; reverse, 5′-GGACTATGTTACAG TGGTCTAGC-3′), β-catenin (forward, 5′-CAGAGTTACTCCACTCCAGGAA-3′; reverse, 5′-CAATGT CCAGTCCAAGATCTGC-3′), cyclin D1 (forward, 5′-CTGGCGCAGGCTTGACTC-3′; reverse, 5′-CATCAAGTGTGACCCGGACT-3′) and cyclin E (forward, 5′-CAGCTTGGATTTGCTGGACAAAG-3′; reverse, 5′-TGTCAGGACCACACTCGGA-3′) were used. The expression data were normalized to the levels of the housekeeping gene and the comparative threshold cycle (2−ΔΔCT) method was used to analyze relative changes.

### 4.8. Statistical Analysis

Quantitative variables are presented as the mean (standard error). Two-tailed *p* < 0.05 was considered statistically significant. Statistical significance between measurements was determined using Student’s *t*-test, Kruskal–Wallis one-way ANOVA, and one-way ANOVA analysis followed by the Duncan multiple-range post hoc test, as appropriate.

## 5. Conclusions

Combination therapy for TNBC using Avastin plus omega-3 polyunsaturated fatty acids and Se showed significantly greater antitumor activities than did Avastin alone, in a preclinical model. The present findings expand our understanding of the effects of using low dose antiangiogenic agent Avastin with nutritional supplements in treating TNBC. We observed the addition of FO and Se to Avastin treatment was crucial to induce: (1) decreases in the phosphorylation of multiple potent proangiogenic (growth) factors and their membrane receptors; (2) regulation of cytoplasmic PI3K-PTEN-AKT-TSC1/2-mTOR-70S6K-4EBP1, Ras-Raf-MEK-ERK, c-Src-JAK2-STAT3-TMEPAI-Smad, LKB1-AMPK, and GSK3β-β-catenin signaling; (3) inhibition of nuclear cyclin and cyclin-dependent kinases; (4) upregulation of tumor apoptosis, as well as; (5) inhibition of the HSP90, HIF-1α/HIF-2α, COX-2, SOD-1, and MMP-9, and; (6) suppression of the EMT and stemness in tumor.

These results suggest that combination treatment with marine-based FO and Se increases the efficacy of low dose Avastin against TNBC via multiple antitumor signaling pathways in a dose-dependent manner and is a potential novel therapeutic strategy for TNBC treatment. Further studies will be needed to evaluate the effects of treatment with the combination of FO and Se with other antiangiogenic agents on TNBC.

## Data Availability

The data is contained within the article.
